# Lowered fasting chenodeoxycholic acid correlated with the decrease of fibroblast growth factor 19 in Chinese subjects with impaired fasting glucose

**DOI:** 10.1038/s41598-017-06252-6

**Published:** 2017-07-20

**Authors:** Jing Zhang, Huating Li, Hu Zhou, Li Fang, Jingjing Xu, Han Yan, Shuqin Chen, Qianqian Song, Yinan Zhang, Aimin Xu, Qichen Fang, Yang Ye, Weiping Jia

**Affiliations:** 1 Shanghai Key Laboratory of Diabetes Mellitus, Department of Endocrinology and Metabolism, Shanghai Diabetes Institute, Shanghai Clinical Center for Diabetes, Shanghai Jiao Tong University Affiliated Sixth People’s Hospital, Shanghai, 200233 China; 2 CAS Key Laboratory of Receptor Research, Shanghai Institute of Materia Medica, Chinese Academy of Sciences, Shanghai, 201203 China; 3 State Key Laboratory of Drug Research, Shanghai Institute of Materia Medica, Chinese Academy of Sciences, Shanghai, 201203 China; 4 Department of Medicine, State Key Laboratory of Pharmaceutical Biotechnology, University of Hong Kong, Hong Kong, 999077 China; 5 Department of Pharmacology and Pharmacy, University of Hong Kong, Hong Kong, 999077 China; 6 Shanghai Key Laboratory of Diabetes Mellitus and Center for Translational Medicine, Shanghai Jiao Tong University Affiliated Sixth People’s Hospital, Shanghai, 200233 China

## Abstract

The gut-derived hormone Fibroblast growth factor 19 (FGF19) could regulate glucose metabolism and is induced by bile acids (BAs) through activating Farnesoid X Receptor (FXR). FGF19 was found to decrease in subjects with isolated-impaired fasting glucose (I-IFG) and type 2 diabetes mellitus (T2DM). However, the reason for the change of FGF19 in subjects with different glucometabolic status remained unclear. Here we measured six BAs including chenodeoxycholic acid (CDCA), cholic acid, deoxycholic acid, their glycine conjugates and FGF19 levels during oral glucose tolerance test (OGTT) in normal glucose tolerance (NGT), isolated-impaired glucose tolerance, I-IFG, combined glucose intolerance (CGI) and T2DM subjects. After OGTT, serum FGF19 peaked at 120 min in all subjects. Glycine conjugated BAs peaked at 30 min, while free BAs did not elevated significantly. Consistent with the decrease trend in FGF19 levels, fasting serum CDCA levels in subjects with I-IFG, CGI and T2DM were significantly lower than NGT subjects (*P* < 0.05). Fasting serum CDCA was independently associated with FGF19. CDCA strongly upregulated FGF19 mRNA levels in LS174T cells in a dose- and time-dependent manner. These results suggest that the decrease of FGF19 in subjects with I-IFG was at least partially due to their decrease of CDCA acting via FXR.

## Introduction

Type 2 diabetes mellitus (T2DM) is a complex metabolic disorder that has been recognized as a challenging contemporary threat to public health^1^. This disease will translate into excess mortality, especially from cardiovascular disease^2^. Pre-diabetes (i.e., impaired fasting glucose (IFG) and/or impaired glucose tolerance (IGT)) represents an intermediate stage between normal glucose tolerance (NGT) and diabetes and is an important risk factor for the development of diabetes^3^. Individuals with IFG have a 20–30% chance of developing diabetes over the next 10 years^4^, and the risk is even greater if they have combined IFG and IGT. Thus, it is essential to explore the pathophysiology of T2DM. Recently, some novel endocrine cytokines have been found to be involved in the pathogenesis of progression from NGT to pre-diabetes, and ultimately to T2DM^5, 6^.

Fibroblast growth factors (FGFs) are a group of proteins that act in autocrine/paracrine or endocrine fashion to regulate various biological processes, such as development, differentiation, and metabolism^7^. FGF21 and FGF19 (also called FGF15 in rodents), members of the FGFs endocrine subfamily, have been shown to exert hormone-like metabolic effects through activation of FGFs receptors^7^. FGF21 is predominantly expressed in the liver and has pleiotropic effects on energy homeostasis and insulin sensitivity through its multiple target organs such as adipose tissue^8^. Patients with pre-diabetes and T2DM showed an increase in serum FGF21 compared with the normal control^9^. FGF19 and its mouse ortholog FGF15 are mainly expressed in the distal small intestine^10, 11^. FGF19 has also been shown to possess potent beneficial effects on glucose metabolism^12^. Transgenic mice expressing FGF19 had increased energy expenditure and improved glucose tolerance^13^. Administration of recombinant FGF19 protein prevented the development of glucose intolerance in both high-fat-fed mice and leptin-deficient mice^14^. FGF15-KO mice had 50% less hepatic glycogen than the wild-type mice^15^. Besides, FGF15-KO mice showed impaired glucose uptake from the circulation, but can be corrected by FGF19 treatment^15^. Studies in mice have demonstrated that FGF19 induced hepatic glycogen synthesis through an insulin-independent Ras-ERK-p90RSK pathway^15^ and repressed gluconeogenesis by inhibiting the activity of the transcription factor cAMP regulatory element binding protein, a key regulator of proliferator-activated receptor γ coactivator-1α (PGC-1α) and other gluconeogenic genes^16^. In humans, decreased fasting FGF19 levels were reported in patients with metabolic syndrome^17, 18^. In our previous study, we found that fasting serum FGF19 levels were reduced in subjects with IFG and T2DM while not in subjects with IGT^6^. Besides, fasting serum FGF19 levels were independently associated with the deterioration of glucometabolic status from NGT to IFG and T2DM^6^. IFG and IGT are two categories of pre-diabetes that have different pathophysiological characteristics of glucose metabolism^3^. IFG is due to increased hepatic glucose production, whereas IGT mainly results from peripheral insulin resistance^3^. These clinical data have provided support for the role of FGF19 as a potential mediator with effects on glucose metabolism in humans^6, 17, 18^. However, the reason for the change of FGF19 in subjects with different glucometabolic status remained unclear.

FGF19 could be upregulated by bile acids (BAs)-mediated activation of Farnesoid X Receptor (FXR)^10, 11^. BAs are a group of structurally diverse molecules synthesized from enzymatic oxidation of cholesterol in the liver, and have long been known to facilitate dietary lipid absorption and regulate cholesterol homeostasis^19^. Recent years, BAs are gaining increasing recognition as important metabolic signaling molecules^19^. Through binding to the G-protein-coupled receptor TGR5 and nuclear receptor FXR, BAs could activate diverse signaling pathways and participate in triglyceride, cholesterol, energy and glucose homeostasis^19^. Studies in animals and humans showed that BAs improved glycemic control^20, 21^. Due to the fact that BAs were involved in glucose homeostasis and FGF19 expression, our aim in this study was to evaluate whether the decrease of FGF19 in subjects with IFG were associated with BAs. To this end, we investigated the physiological change of serum FGF19 and individual BAs following 75-g oral glucose tolerance test (OGTT) in Chinese subjects with different glucose tolerance categories, and explored the relationship between serum individual BAs and FGF19.

## Results

### Characteristics of study subjects

The clinical characteristics of 245 subjects with NGT, isolated-impaired glucose tolerance (I-IGT), isolated-impaired fasting glucose (I-IFG), combined glucose intolerance (CGI) and newly diagnosed T2DM were shown in Table 1. No significant differences in age, gender, body mass index (BMI) and blood pressure were observed among these subjects. As expected, individuals with T2DM were hyperglycemic, with higher fasting plasma glucose concentration (FPG) and 2-h plasma glucose concentration (2hPG) (all *P* < 0.001). These variables were intermediate in pre-diabetes. Fasting serum insulin concentration (FINS) in T2DM group were higher than in NGT group (*P* < 0.01). Subjects with I-IFG, CGI and T2DM had elevated HOMA-IR in comparison with NGT (all *P* < 0.05).

**Table 1:** Anthropometric parameters and biochemical indexes among study subjects (n = 245).

Variables	NGT (n = 63)	I-IGT (n = 37)	I-IFG (n = 30)	CGI (n = 37)	T2DM (n = 78)	*P*
Male/Female (n)	36/27	20/17	15/15	18/19	39/39	0.902
Age (years)	53.7 ± 11.7	54.0 ± 12.8	52.9 ± 13.3	54.0 ± 8.9	55.7 ± 12.9	0.870
BMI (kg/m^2^)	23.5 ± 2.7	24.1 ± 3.0	23.8 ± 2.3	24.3 ± 2.3	24.3 ± 3.1	0.470
SBP (mmHg)	123.92 ± 15.62	126.89 ± 15.81	130.70 ± 16.90	131.65 ± 21.37	129.35 ± 18.20	0.183
DBP (mmHg)	74.68 ± 9.68	76.43 ± 10.53	73.77 ± 11.99	77.68 ± 12.78	77.19 ± 9.84	0.381
FPG (mmol/L)^§^	5.25 (4.84–5.56)	5.47 (5.04–5.71)	6.38^‡^ (6.20–6.60)	6.38^‡^ (6.27–6.68)	7.11^‡^ (6.33–7.51)	<0.001
2hPG (mmol/L)^§^	6.23 (5.55–6.88)	9.31^‡^ (8.19–10.01)	6.55 (5.84–7.20)	9.55^‡^ (8.94–10.22)	13.78^‡^ (10.70–16.03)	<0.001
FINS (μU/mL)^§^	6.60 (5.02–10.09)	8.78 (5.89–11.68)	9.01 (6.01–10.79)	6.56 (5.66–8.53)	10.13^‡^ (6.77–12.38)	0.001
2hINS (μU/mL)^§^	56.86 (35.99–92.40)	102.20^‡^ (56.65–143.40)	65.51 (33.58–88.61)	69.23 (47.22–94.28)	76.78^†^ (51.77–111.20)	<0.001
HOMA-IR^§^	1.53 (1.17–2.34)	2.18 (1.30–2.83)	2.57^‡^ (1.74–2.99)	1.92^†^ (1.63–2.45)	3.10^‡^ (2.12–4.01)	<0.001
HOMA-%B^§^	80.00 (55.44–112.20)	92.89 (65.95–127.06)	61.52^‡^ (39.68–81.39)	43.85^‡^ (38.02–58.73)	60.41^‡^ (36.86–84.04)	<0.001

Data are mean ± SD or median (interquartile range). ^§^Log transformed before analysis. CGI, combined glucose intolerance; DBP, diastolic blood pressure; FINS, fasting serum insulin concentration; FPG, fasting plasma glucose concentration; HOMA-%B, homeostasis model assessment of insulin secretion; HOMA-IR, homeostasis model assessment of insulin resistance; I-IFG, isolated-impaired fasting glucose; I-IGT, isolated-impaired glucose tolerance; NGT, normal glucose tolerance; 2hPG, 2-h plasma glucose concentration; SBP, systolic blood pressure; T2DM, type 2 diabetes mellitus; 2hINS, 2-h serum insulin concentration; ^†^ *P* < 0.05, compared with NGT; ^‡^ *P* < 0.01, compared with NGT.

### Fasting and postload serum FGF19 during OGTT in subjects with different glucose tolerance status

**Figure 1:**
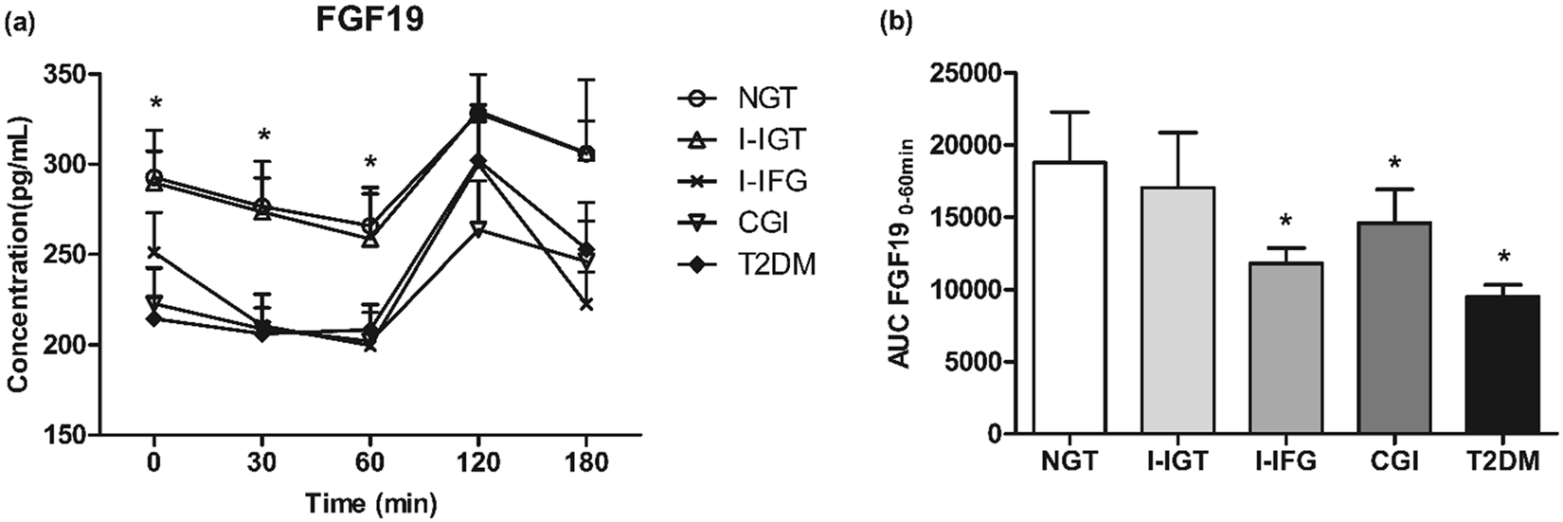
Fasting and postload FGF19 concentration (n = 245). (**a**) Dynamic change of serum FGF19 in different glucose tolerance status after OGTT. *P < 0.05 among the five groups. (**b**) AUC-FGF19_0–60 min_ in different glucose tolerance status. *P < 0.05 compared with NGT. Data are mean ± SEM.

We first investigated the fasting and postload serum FGF19 levels during OGTT in subjects with different glucose tolerance status (Fig. 1a). Serum FGF19 concentration peaked at 120 min, but significant differences were observed in serum FGF19 levels at 0, 30 and 60 min rather than at 120 and 180 min among the five groups. Fasting as well as 30 and 60 min serum FGF19 levels in subjects with I-IFG, CGI and T2DM were lower than those in NGT subjects (*P* < 0.05). However, no significant difference in serum FGF19 levels was observed between I-IGT and NGT subjects at 0, 30 and 60 min. When the serum FGF19 levels from 0 to 60 min were expressed as area under the curve (AUC-FGF19_0–60 min_), significant decrease were also found in subjects with I-IFG, CGI and T2DM in comparison with the healthy controls (Fig. 1b). AUC-FGF19_0–60 min_ was inversely correlated with AUC-PG_0–60 min_ (*r* = −0.197, *P* = 0.002). Multiple stepwise regression analysis involved BMI, FGF19 0 min, FGF19 30 min, FGF19 60 min, FINS, insulin 30 min and insulin 60 min revealed that FGF19 0 min (standard β = −0.125, t = −2.185, *P* = 0.030), FINS (standard β = 0.367, t = 5.853, *P* < 0.001) and insulin 30 min (standard β = −0.463, t = −7.461, *P* < 0.001) were independently associated with AUC-PG_0–60 min_, suggesting that fasting FGF19 played important roles in glucose homeostasis.

### Profiles of serum BAs during OGTT in subjects with different glucose tolerance status

**Figure 2:**
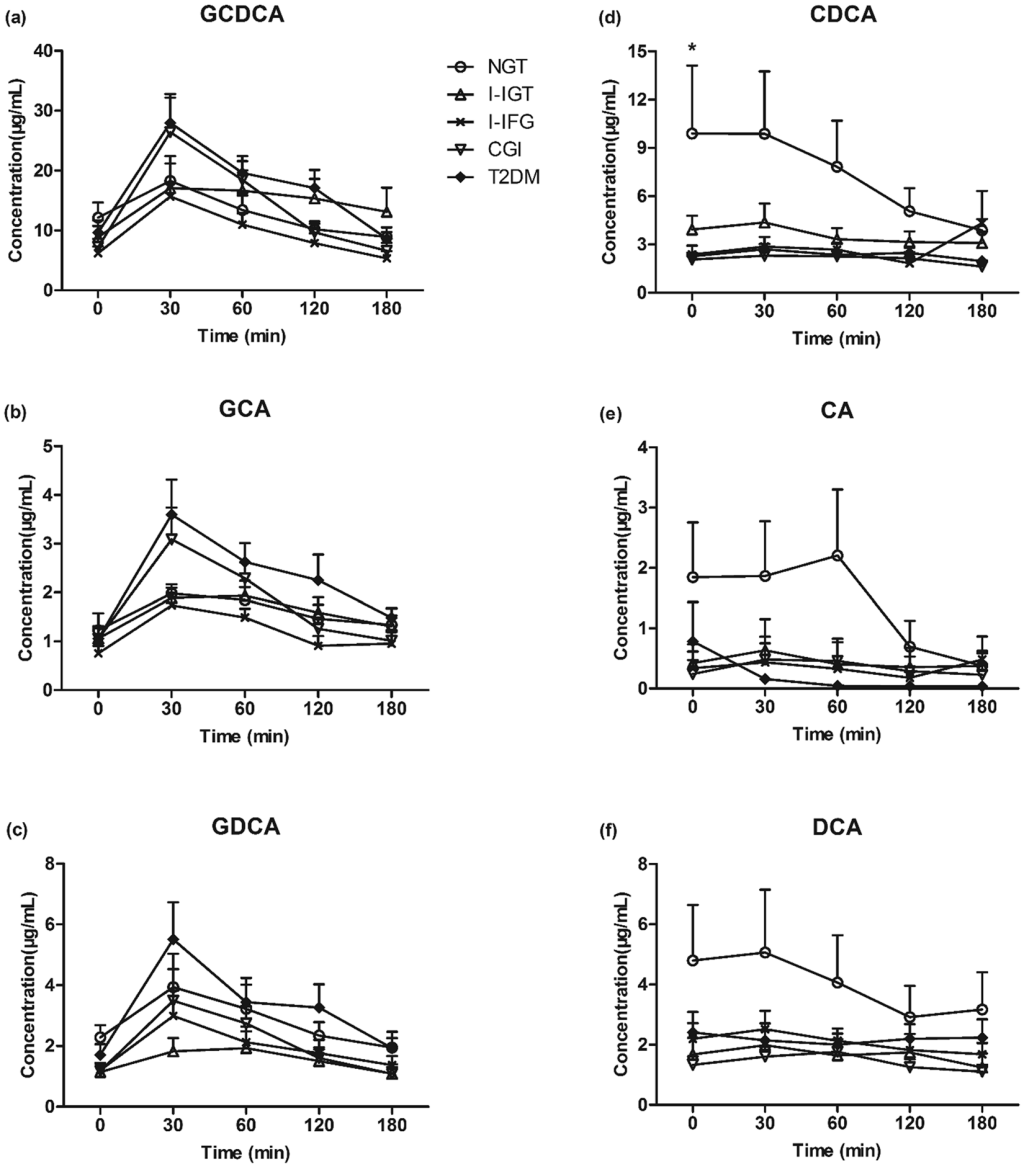
Dynamic change of serum GCDCA (**a**), GCA (**b**), GDCA (**c**), CDCA (**d**), CA (**e**) and DCA (**f**) in different glucose tolerance status after OGTT (n = 65). *P < 0.05 among the five groups.

As FGF19 expression could be induced by BAs-mediated activation of the FXR, we selected 10 NGT, 9 I-IGT, 10 I-IFG, 12 CGI and 24 T2DM subjects that were matched in terms of sex, age and BMI as a subgroup to investigate profiles of BAs. We measured serum levels of BAs including chenodeoxycholic acid (CDCA), cholic acid (CA), deoxycholic acid (DCA) and their glycine conjugates at baseline, 30, 60, 120 and 180 min after the glucose load. No significant difference was found in serum total BAs, G-BAs, free BAs and individual BAs levels between male and female subjects. As shown in Fig. 2, the serum kinetics of glycine-conjugated BAs (G-BAs) including glycochenodeoxycholic acid (GCDCA), glycocholic acid (GCA) and glycodeoxycholic acid (GDCA) showed a uniform pattern characteristic in all the five groups. G-BAs levels increased to a peak at 30 min (*P* < 0.05 vs 0 min) and significantly decreased over the next 150 min (*P* < 0.05 vs 30 min). Different from G-BAs, serum concentration of free BAs including CDCA, CA and DCA decreased or remained unchanged in response to oral glucose challenge.

### Differences of serum BAs concentration and compositions in subjects with different glucose tolerance status

**Figure 3:**
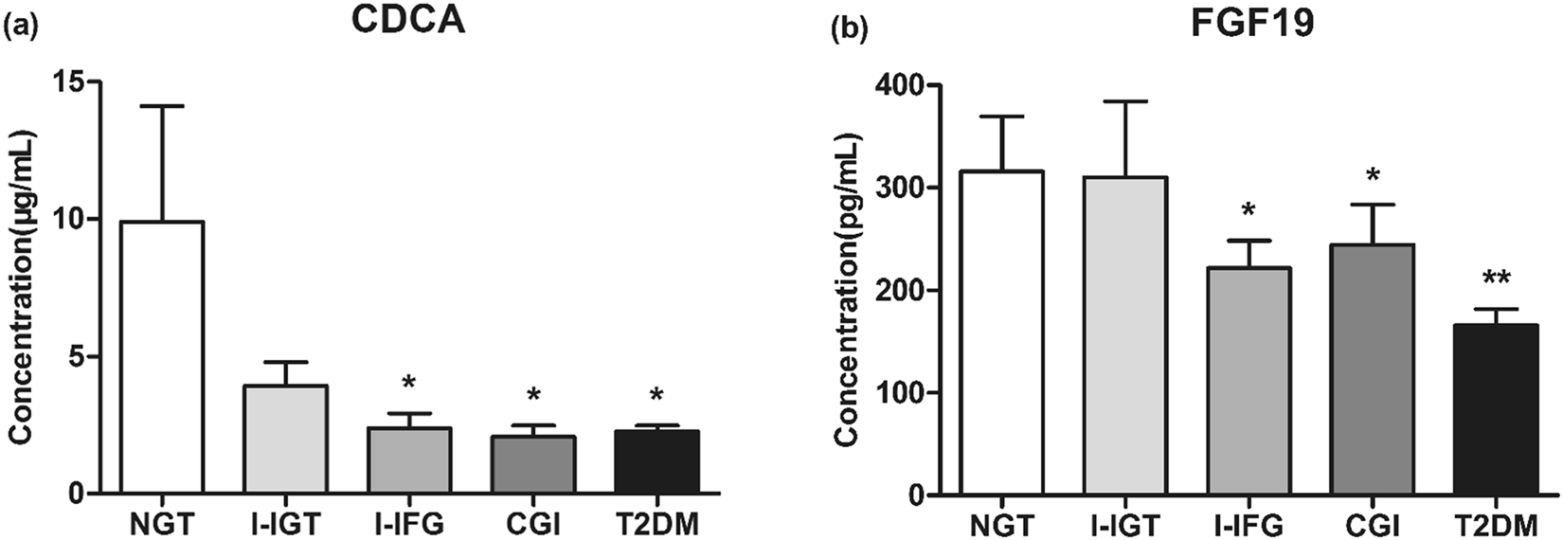
Fasting serum CDCA (**a**) and FGF19 (**b**) concentration in subjects with NGT (n = 10), I-IGT (n = 9), I-IFG (n = 10), CGI (n = 12) and T2DM (n = 24). *P < 0.05, vs. NGT; **P < 0.01, vs. NGT.

We further compared serum BAs levels among the five groups during OGTT. Both fasting and postprandial concentration of total BAs, total glycine-conjugated BAs and total free BAs were similar in all the five groups (Supplementary Fig. S1). Regarding individual BAs at 0, 30, 60, 120 and 180 min, no significant alterations of the serum G-BAs including GCDCA, GCA and GDCA and free BAs including DCA and CA were observed among all subjects (Supplementary Figs S2, S3). However, fasting serum CDCA differed across the five groups (*P* = 0.043), with lower concentration in subjects with I-IFG, CGI and T2DM than NGT (all *P* < 0.05), while no significant difference in fasting serum CDCA levels was observed between I-IGT and NGT subjects (Fig. 3a). There were no significant differences in CDCA levels at 30, 60, 120 and 180 min after OGTT among the five groups (Supplementary Fig. S3). We also investigated the specific proportions of BAs associated with different glucose tolerance state. As shown in Supplementary Fig. S4, in all subjects, the proportion of fasting CDCA showed a trend of decrease in I-IFG (18%), CGI (16%) and T2DM (13%) compared with NGT (31%).

**Figure 4:**
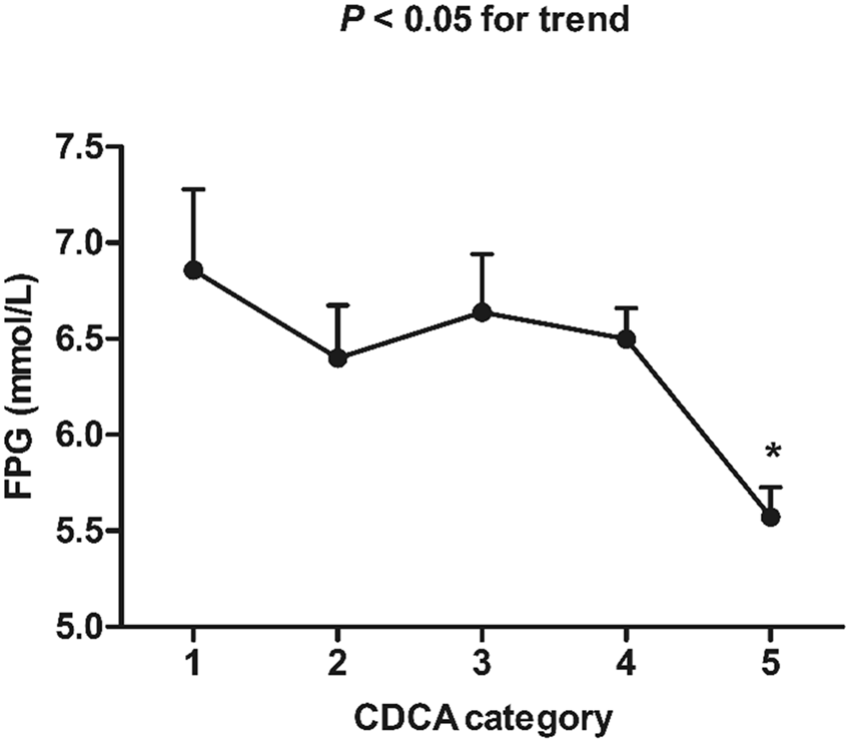
Comparison of FPG between different CDCA categories. CDCA categories are defined as follows: category 1 (fasting CDCA concentration <1.0 μg/mL, n = 10), category 2 (fasting CDCA concentration: 1.0–2.0 μg/mL, n = 15), category 3 (fasting CDCA concentration: 2.0–3.0 μg/mL, n = 13), category 4 (fasting CDCA concentration: 3.0–4.0 μg/mL, n = 16), category 5 (fasting CDCA concentration ≥4.0 μg/mL, n = 11). *P < 0.05, compared with the first CDCA category.

A Spearman’s correlation was performed to investigate the relationships between the individual BAs at the five time points during OGTT and a cluster of anthropometric parameters, biochemical indexes, insulin secretion and sensitivity. The analysis demonstrated an inverse association of fasting CDCA with FPG (r = −0.249, *P* = 0.049). We have compared fasting CDCA levels between different glucose tolerance states. To further compare FPG between different CDCA categories, fasting CDCA were then divided in five categories according to the fasting serum concentration of CDCA: category 1 (fasting CDCA concentration <1.0 μg/mL, n = 10), category 2 (fasting CDCA concentration: 1.0–2.0 μg/mL, n = 15), category 3 (fasting CDCA concentration: 2.0–3.0 μg/mL, n = 13), category 4 (fasting CDCA concentration: 3.0–4.0 μg/mL, n = 16), category 5 (fasting CDCA concentration ≥4.0 μg/mL, n = 11) (Fig. 4). FPG showed a decrease trend in the five categories. Compared to the first category, FPG in the fifth category was significantly lower (*P* < 0.05).

### Fasting serum CDCA levels were positively associated with FGF19

**Figure 5:**
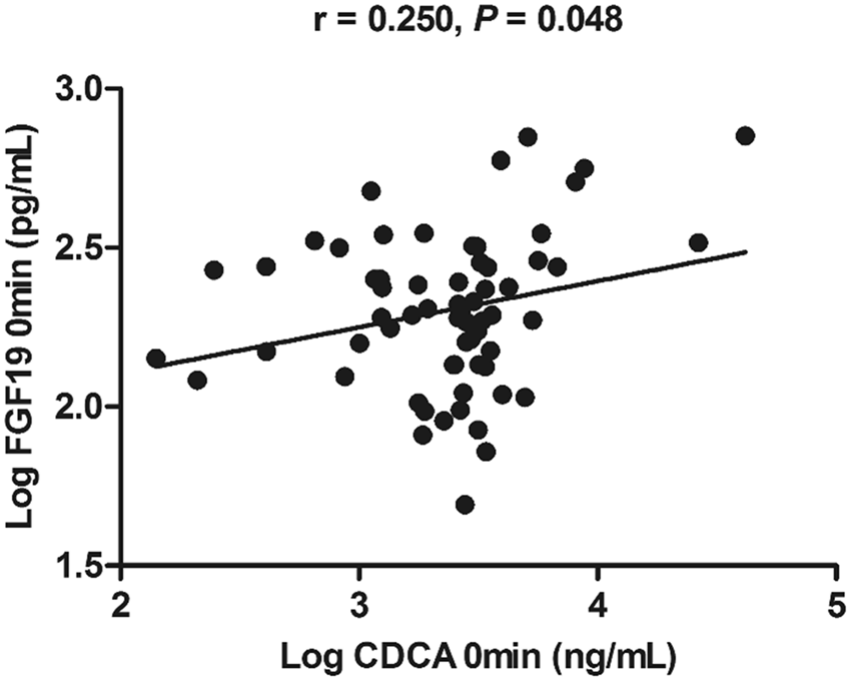
Correlations of serum levels of CDCA (log transformed) with FGF19 (log transformed) (n = 65).

We next determined bivariate correlations of serum FGF19 with BAs compositions and glycemic measures in the subgroup. Fasting serum FGF19 levels were decreased in Chinese subjects with I-IFG, CGI and T2DM (Fig. 3b), and were negatively associated with FPG (r = −0.260, *P* = 0.036). Neither fasting nor postload FGF19 correlated with total BAs, total free or total glycine-conjugated BAs. In contrast, fasting serum FGF19 levels were found to be positively related with the corresponding CDCA levels (r = 0.250, *P* = 0.048) (Fig. 5).

### FGF19 was induced by the FXR agonist CDCA in intestinal cells

**Figure 6:**
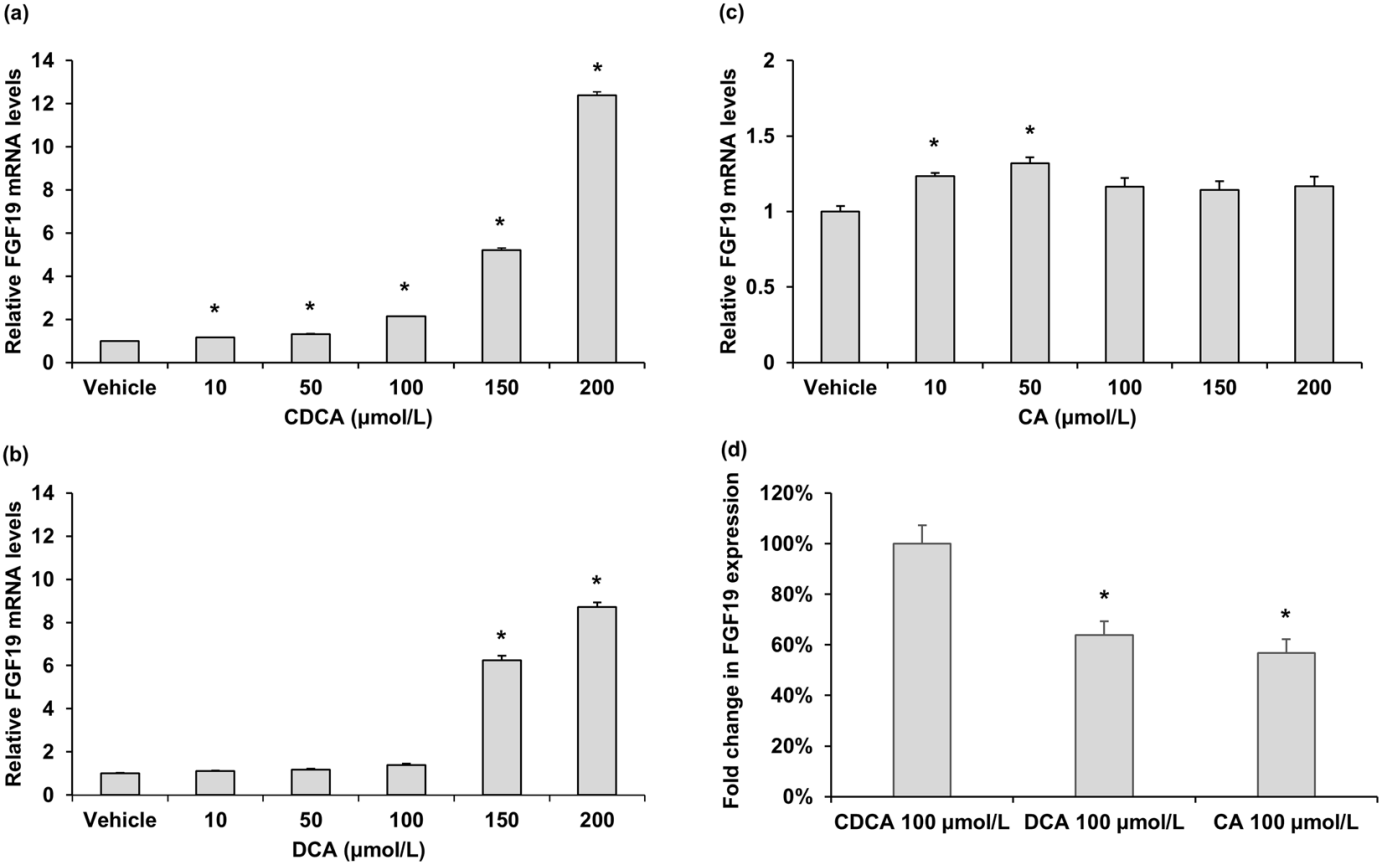
Effect of BAs treatment on FGF19 mRNA levels in LS174T cells. Effect of different concentration of CDCA (**a**), DCA (**b**) and CA (**c**) treatment on FGF19 mRNA levels. Data are mean ± SEM, n = 4. *P < 0.05, compared vs. vehicle. (**d**) Relative potencies of 100 μmol/L CDCA, DCA and CA at inducing FGF19 mRNA expression in LS174T cells. Data are mean percentages ± SEM, n = 4. *P < 0.05, compared vs. CDCA 100 μmol/L.

**Figure 7:**
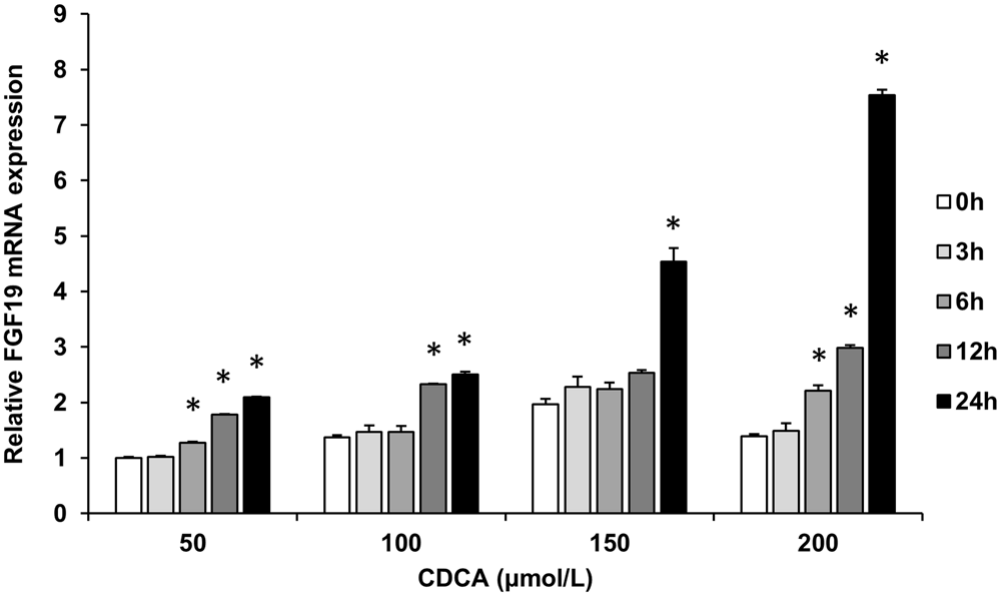
Time course of the effect of different concentration CDCA on FGF19 mRNA levels in LS174T cells. The level of FGF19 mRNA in cells incubated with 50 μmol/L CDCA for 0 h was set at 1, and the other values were adjusted proportionately. Data are mean ± SEM, n = 3. *P < 0.05, compared vs. 0 h of respective concentration.

To further examine the BAs-induced regulation of FGF19, human epithelial colon cell line LS174T was cultured in the presence of various concentration of BAs. Stimulation of LS174T cells with CDCA resulted in a significant induction of FGF19 expression, starting at lower concentration but most prominently after 24 h at a concentration of 200 μmol/L (Fig. 6a). These data clearly demonstrated that the FGF19 gene could be activated by the FXR ligand CDCA. Incubating LS174T with DCA also stimulated a dose-dependent increase in FGF19 mRNA levels (Fig. 6b). Whereas treatment with CA had no dose-dependent increase in FGF19 mRNA levels (Fig. 6c). After treatment with 100 μmol/L CDCA in parallel with the same concentration of DCA and CA, relative induction of FGF19 mRNA was expressed as a percentage of the induction observed in LS174T cells stimulated with 100 μmol/L CDCA. The mean induction of FGF19 mRNA expression in LS174T cells incubated with 100 μmol/L DCA was 64% of that seen with respective paired CDCA incubations, whereas lower induction was found with 100 μmol/L CA (56%) (Fig. 6d). The data suggested the potency of BAs to stimulate FGF19 expression is CDCA > DCA > CA. We further investigated the time dependent influence of CDCA on FGF19 expression. LS174T cells were stimulated for 0, 3, 6, 12 and 24 h with four different concentrations (50, 100, 150 and 200 μmol/L) of CDCA. Time-course experiments showed that CDCA time-dependently induced FGF19 expression (Fig. 7).

### The association between CDCA and FPG was partially dependent on FGF19

We then performed multiple stepwise regression analysis to determine which parameters were independently associated with serum FGF19. The analysis involved age, CDCA, DCA and CA. Fasting serum CDCA (standard β = 0.496, t = 2.354, *P* = 0.031) was found to be independently associated with fasting serum FGF19. As we have demonstrated the negative association between fasting serum CDCA levels with FPG, partial correlation analysis was performed to investigate whether FGF19 was involved in the relationship between fasting CDCA and FPG. Notably, the association between CDCA and FPG was no longer significant after adjustment for FGF19 levels (r = −0.218, *P* = 0.089).

### CDCA increased the effect of FGF19 in HepG2 cells

Previous study has reported that FGF15/19 repressed PGC-1a and its target genes glucose-6-phosphatase (G6Pase), which encode proteins involved in gluconeogenesis^16^. To further investigate CDCA increasing the effect of FGF19, HepG2 cells were treated with CDCA in the presence or absence of FXR antagonist guggulsterone (GS). CDCA alone induced FGF19 expression significantly, and treatment of HepG2 cells with CDCA together with GS inhibited the CDCA-induced FGF19 expression (Supplementary Fig. S5a). CDCA alone downregulated the expression of PGC-1α and G6Pase. While in the presence of GS, the repression of PGC-1α and G6Pase expression by CDCA no longer existed (Supplementary Fig. S5b,c). As FGF19 could be upregulated by CDCA-mediated activation of FXR, these results suggested that CDCA could increase the effect of FGF19.

## Discussion

In addition to their well-established roles in cholesterol homeostasis, BAs also behave as signaling molecules participating in glucose homeostasis and FGF19 expression^19^. In this study, we highlighted the link of CDCA with FGF19 in Chinese subjects with different degrees of glucose intolerance. Fasting serum CDCA levels decreased in I-IFG, CGI and T2DM subjects, which was coincided with the decrease trend in FGF19 concentration. Our results suggested the possibility that the change of fasting FGF19 in subjects with different glucometabolic status might be related with BAs.

After meals, BAs levels are tightly regulated, and FGF19 is part of the regulators that is secreted in response to the transintestinal flux of BAs and signals from the intestine to the liver to regulate BAs homeostasis^12^. In the investigation by T. Lundasen *et al*., normal volunteers participated in the study, and fasting and postprandial serum FGF19 and total BAs levels were analyzed. When regular meals were ingested, FGF19 peak in serum about 1.5–3 h following the peak of serum BAs, and the subsequent reduction of BA synthesis was observed. When subjects were fasting overnight and the following day, serum BAs gradually declined with time, and serum FGF19 levels did not change, the subsequent reduction of BA synthesis when food was regularly ingested was less evident^22^. In our study, a significant increase at 30 min after glucose intake was observed for glycine species in all the subjects, which might be because BAs are predominantly conjugated with glycine to become glycine conjugates in humans after synthesis in the liver in response to ingestion^23, 24^. Notably, we found that serum FGF19 concentration peaked at 120 min after oral glucose load in the pre-diabetic and diabetic subjects as well as the NGT subjects. However, the peak levels of FGF19 were not significantly different among groups with different glucometabolic status. In the fasting state, serum FGF19 levels were negatively associated with FPG^6^. Significant difference was found in FGF19 levels in the fasting state among the different glucose tolerance categories^6^. Moreover, fasting serum FGF19 but not serum FGF19 at 30 and 60 min was found to be independently associated with AUC-PG_0–60 min._ These results further suggested that fasting FGF19 played an important role in glucose homeostasis; and the postload increase of FGF19 could be attributed to the postprandial BAs release and serum FGF19 elevated to exert feedback control of hepatic BA homeostasis^22^.

Prior animal and clinical studies have demonstrated alterations in BAs compositions between normal control and diabetes^25, 26^. In Hassan *et al*. study, the pool of CDCA was significantly decreased in diabetic rats^26^. Uchida *et al*. also reported decreases in BAs derived from CDCA, such as beta-muricholic and ursodeoxycholic acids, after the development of diabetes in spontaneously diabetic female mice^27^. Clinical investigations showed that diabetic patients had lower percent of CDCA than the normal control^25^. However, in those studies, the change of BAs levels in subjects with pre-diabetes including I-IFG, I-IGT and CGI remained unclear. Using a targeted metabonomics approach, we first demonstrated that subjects with I-IFG, CGI and T2DM had decreased fasting serum CDCA levels than the NGT subjects, but no significant difference in CDCA levels was observed between I-IGT and NGT subjects. The pathophysiology relevance between CDCA and different glucose tolerance categories is reminiscent of the results in our previous and present studies that fasting serum FGF19 levels were decreased in subjects with I-IFG and T2DM but not in I-IGT^6^. Animal-based studies have provided evidence for the role of FGF19 in inducing hepatic glycogen synthesis and inhibiting gluconeogenesis. The change of FGF19 levels in I-IFG subjects further demonstrated the plausible physiological roles for FGF19 in regulating hepatic glucose production^6^, but the reason for the decreased serum FGF19 remained unknown. The similar trend of fasting serum concentration of CDCA and FGF19 in NGT, I-IFG and T2DM raised the possibility that the change of FGF19 levels in subjects with different glucose tolerance state might be related with CDCA.

In this study, we found a positive correlation of fasting serum CDCA levels with FGF19, and treatment of human epithelial colon cell line LS174T cell with CDCA caused a dose- and time-dependent increase in FGF19 mRNA levels. Besides, the significant association of CDCA with FPG no longer existed after adjustment for FGF19 levels. These findings suggested that CDCA may have the potential to explain the change of FGF19 levels in subjects with different glucose tolerance state. BAs synthesis shows a strong diurnal rhythm, which is entrained by starvation and feeding as well as nutrient status^28, 29^. Circulating FGF19 levels exhibit a pronounced circadian rhythm controlled by the transintestinal BAs flux^22^. Changes in BAs concentration or compositions may alter their ability to activate FXR, and therefore have potential to affect their effects on metabolism. CDCA has been used as medical therapy to treat gallstones^30^ and cerebrotendineous xanthomatosis^31^. DCA was found to promote glucagon-like peptide-1 secretion^32^, leading to improved liver and pancreatic function and enhanced glucose tolerance^33^. Previous study pointed towards the effect of DCA as an immunostimulant^34^. CA was found to prevent hepatic triglyceride accumulation, VLDL secretion, and elevated serum triglycerides^35^. The hydrophobic CDCA was the most efficacious endogenous FXR ligand, while DCA activated FXR with a lower efficacy than CDCA, and CA had negligible activities^36^, which were consistent with our result that the potency of BAs to stimulate FGF19 expression is CDCA > DCA > CA. Compared with DCA and CA, CDCA was found to have the highest potential to activating FXR in LS174T cells^37^. A previous clinic study found that intraduodenal infusion of CDCA could result in a dose-dependent rise in plasma FGF19 concentration^38^. In another study, patients with T2DM and nonalcoholic fatty liver disease treatment with semisynthetic derivative of CDCA led to enhanced serum FGF19 levels and improved insulin sensitivity^39^. Moreover, our colleagues recently reported that increased CDCA was correlated with a shorter duration of T2DM, which was associated with a higher possibility of remission after Roux-en-Y Gastric Bypass surgery^40^. Therefore, our finding that serum CDCA levels were independently correlated with FGF19 levels demonstrated the plausible physiological roles for CDCA in regulating FGF19 expression, and further suggested that the decrease of FGF19 in subjects with elevated FPG might be at least partially associated with their decrease of CDCA acting via FXR.

The decrease of fasting serum CDCA levels in subjects with elevated FPG might be attributed to altered gut microbial compositions. BAs in humans include the primary BAs, CA and CDCA, and their respective secondary BAs, DCA and trace amount of lithocholic acid (LCA), which are formed via deconjugation and 7-dehydroxylation by enzymes in gut bacteria^41^. Importantly, 7-dehydroxylation activity was not detectable for glycine or taurine conjugated primary BAs^42^. Dehydroxylation appears restricted to free BAs^43^. No 7-dehydroxylation was observed when the conjugated BAs were incubated with *Eubacterium* sp. that can 7-dehydroxylate BAs^43^. While incubation of BAs with *Eubacterium* sp. together with *C.perfringens*, a bacterial species that could hydrolyze BAs conjugates, resulted in 7-dehydroxylation^43^. Removal of glycine/taurine BAs conjugates is thus a prerequisite for 7-dehydroxylation by intestinal bacteria. Bacterial bile salt hydrolase are enriched in the human gut microbiome, and they are able to catalyze the deconjugation of conjugated BAs to generate unconjugated BAs^44^. It was reported that some of these hydrolases were active in intestinal microflora such as *Lactobacillus* ^45^ and *Bacteroides* ^46^, which were found to be reduced in the gut microbial environment of diet-induced diabetic mice or *ob/ob* mice^47^. Thus, the decreased levels of unconjugated CDCA in subjects with elevated FPG could potentially be caused by the reduction of bacteria, which led to lower rates of BAs deconjugation in the intestine.

There are several limitations in the present study. The sample size was relatively small when we investigated profiles of serum BAs in the subgroup. Ample sample sizes are needed to further confirm the relationship between serum CDCA and FGF19 in subjects with different glucometabolic status. Additional experiments are also required to carefully define the CDCA-increasing effects of FGF19. Second, our study design was cross-sectional and did not address the cause-effect relationship between CDCA and I-IFG, CGI as well as T2DM. Further prospective studies are warranted to determine whether decreased serum CDCA is causally related to the exceeded FPG.

In summary, this study described the response of serum FGF19 and BAs to the glucose challenge in different glucose tolerance status. Fasting serum CDCA levels were decreased in I-IFG, CGI and T2DM subjects, which was coincided with the decrease trend in FGF19 concentration. Our data demonstrated that serum concentration of fasting serum CDCA were independently correlated with FGF19, and CDCA could strongly up-regulate FGF19 mRNA levels in LS174T cells in a dose- and time-dependent manner. The significant association between CDCA and FPG no longer existed after adjustment for FGF19 levels. These results suggested that the decrease of FGF19 in subjects with elevated FPG was at least partially due to their decrease of CDCA acting via FXR.

## Methods

### Subjects

A total of 245 individuals with different glucometabolic status were enrolled from the Department of Endocrinology and Metabolism in Shanghai Jiao Tong University Affiliated Sixth People’s Hospital from January 2011 to August 2012. OGTT was performed among these subjects. The diagnosis of various glucose tolerance status was based on the 2003 American Diabetic Association diagnostic criteria^48^ and the definition of I-IGT and I-IFG were based on Meyer *et al*. study^3^. FPG < 6.1 mmol/L and 2hPG < 7.8 mmol/L were classified as NGT. I-IGT was defined as a 2hPG of 7.8–11.1 mmol/L and normal FPG. I-IFG was defined as a FPG of 6.1–7.0 mmol/L and normal 2hPG. CGI was defined as a FPG of 6.1–7.0 mmol/L and 2hPG of 7.8–11.1 mmol/L. Among all subjects, 63 had NGT, 37 had I-IGT, 30 had I-IFG, 37 had CGI and 78 had T2DM. Serum BAs concentration were measured in a subgroup of 65 individuals with different glucometabolic status. Among them, 10 had NGT, 9 had I-IGT, 10 had I-IFG, 12 had CGI and 24 were T2DM. All the participants underwent comprehensive physical examinations, routine biochemical analyses of blood and electrocardiogram. The participants completed a uniform questionnaire containing questions about the histories of present and past medical therapy. Subjects with the following conditions were excluded from this study: acute infectious disease, biliary obstructive diseases, alcoholic abuse, acute or chronic cholecystitis, acute or chronic virus hepatitis, cirrhosis, diarrhea, known hyperthyroidism or hypothyroidism, chronic renal insufficiency, heart failure, presence of cancer, pregnancy and stroke in acute phase, current treatment with BAs and BAs sequestrant that may affect BAs metabolism, and drugs that effect insulin secretion and sensitivity. The study complied with the Declaration of Helsinki and was approved by the ethics committee of Shanghai Jiao Tong University Affiliated Sixth People’s Hospital. All the subjects gave informed consent.

### Clinical and Biochemical measurements

BMI was calculated as weight (kg)/height (m^2^). Systolic blood pressure and diastolic blood pressure were also measured. Blood samples were obtained at 0, 30, 60, 120 and 180 min during OGTT. PG levels were quantified by the hexokinase method. Serum levels of insulin were assayed by radioimmunoassay (Linco Research, St. Charles, MO). Basal insulin secretion and insulin sensitivity were assessed by homeostasis model assessment of insulin secretion (HOMA-%B) and homeostasis model assessment of insulin resistance (HOMA-IR). HOMA-%B = [FINS (mU/L) × 6 × 3.33]/[FPG (mmol/L) − 3.5]. HOMA-IR = FINS (mU/L) × FPG (mmol/L)/22.5^49^. Serum FGF19 levels were determined using the enzyme-linked immunosorbent assay (ELISA) kits (Antibody and Immunoassay Services, University of Hong Kong). The assay has been validated in our previous study and was proven to be highly specific to human FGF19^6^. The intra- and inter-assay variations were 4.7 and 5.6%, respectively.

### Quantification of serum BAs by liquid chromatography-mass spectrometry (LC-MS) methods

BAs are predominantly conjugated to glycine in humans. Reference standards of six BAs were acquired from Sigma-Adrich (St. Louis, MO, USA), including CDCA, CA, DCA and the glycine conjugated species GCDCA, GCA, GDCA. Deuterated internal standard (IS) cholic acid-2,2,4,4-D4 was obtained from C/D/N Isotopes Inc (Quebec, Canada).

Serum BAs concentration were measured at baseline, 30, 60, 120 and 180 min after the oral glucose load. Sample preparation for BAs LC-MS analysis followed the protocol reported by Scherer *et al*.^50^, with modifications. Briefly, BAs were extracted from 50 μL of serum mixed with 100 μL of acetonitrile (contains IS). After centrifugation, the supernatant was dried under a nitrogen stream and resuspended in 50 μL of methanol. Followed by an additional centrifugation, the methanolic supernatant was used for LC-MS analysis. The LC-MS system consisted of an Agilent 1290 Infinity Ultra high-performance liquid chromatography (UHPLC), an electrospray ionization source with Agilent Jet Steam Technology and an Agilent Triple Quadrupole Mass Spectrometer (G6460A) using multiple-reaction monitoring negative ion mode (Agilent Technologies, CA, USA). Liquid chromatography was performed on the Agilent 1290 Infinity UHPLC with a Waters Atlantis T3 column (2.1 mm × 150 mm, 3 μm), using methanol and water (containing 0.05% acetic acid) as the mobile phases.

Total BAs was defined as the summation of the 6 individual BAs. Total glycine-conjugated BAs was defined as the summation of GCDCA, GCA and GDCA. Total free BAs was defined as the summation of CDCA, CA and DCA.

### Cell culture and BAs stimulation experiments

The human intestinal cell line LS174T and human hepatoma cell line HepG2 were used for BAs stimulation experiments. Cells were grown in MEM containing 10% fetal bovine serum and penicillin-streptomycin. Cultures were maintained at 37 °C in a humidified 5% CO_2_ atmosphere. The physiological concentrations of CDCA is 10–25 μmol/L. For BAs stimulation experiments, LS174T cells were stimulated for 24 h with five different concentration (10, 50, 100, 150 and 200 μmol/L) of CDCA, CA, DCA (Sigma) or vehicle (0.1% DMSO) in the absence of fetal bovine serum. For the time-course experiments, LS174T cells were stimulated for 0, 3, 6, 12 and 24 h with four different concentrations (50, 100, 150 and 200 μmol/L) of CDCA in the absence of fetal bovine serum. For CDCA increasing the effect of FGF19, HepG2 cells were stimulated for 24 h with 100 μmol/L of CDCA in the presence or absence of 10 μmol/L of FXR antagonist guggulsterone (TOCRIS Bioscience, United Kingdom).

### Quantitative Real time PCR (RT-PCR) Analysis

Total RNA was prepared from LS174T cells and HepG2 cells using the Trizol reagent (ambion). RT-PCR was performed with a Roche Lightcycler 96 system, using the FastStart Universal SYBR Green Master (ROX) (Roche). GAPDH was used as a control. Relative mRNA levels were calculated by the comparative threshold cycle method. The primer sequence sets used were as follows: FGF19, sense, 5′-CAATGTGTACCGATCCGAGAAG-3′ and antisense, 5′-GGGCAGGAAATGAGAGAGT GG-3′; PGC-1α, sense, 5′-AACAGCAGCAGAGACAAATGCACC-3′ and antisense 5′-TGCAGTT CCAGAGAGTTCCACACT-3′; G6Pase, sense, 5′-GGGAAAGATAAAGCCGACCTAC-3′ and antisense 5′-CAGCAAGGTAGATTCGTGACAG-3′; GAPDH, sense, 5′-GGGAAGGTGAAGGTC GGAGT-3′ and antisense, 5′-TTGAGGTCAATGAAGGGGTCA-3′.

### Statistical analysis

SPSS version 16.0 (SPSS, Inc, Chicago, IL) was applied to statistical analysis of the data obtained in this study. Normally distributed data were expressed as mean ± SD. Data that were not normally distributed, as determined by using the Shapiro-Wilk test, were logarithmically transformed before analysis and expressed as median with interquartile range (IQR). Comparisons between different time points were carried out by using paired t test. One-way analysis of variance was used as appropriate for comparisons between groups. Pearson correlations were performed to assess the relationships between serum BAs and anthropometric and biochemical variables. Multiple stepwise regression analysis was used to examine the association of serum FGF19 and other parameters. Two-tailed *P*-values < 0.05 were considered significant.

## Electronic supplementary material

supplementary figure
