# Supplementary material for: Lowered fasting chenodeoxycholic acid correlated with the decrease of fibroblast growth factor 19 in Chinese subjects with impaired fasting glucose
Source: Sci Rep. 2017 Jul 20;7:6042. doi: 10.1038/s41598-017-06252-6 (PMC5519593; doi:10.1038/s41598-017-06252-6)

Lowered fasting chenodeoxycholic acid correlated with the decrease of fibroblast growth factor 19 in Chinese subjects with impaired fasting glucose

Jing Zhang [1], Huating Li [1], Hu Zhou [2], Li Fang [2], Jingjing Xu [2],
Han Yan [1], Shuqin Chen [1], Qianqian Song [1], Yinan Zhang [6], Aimin Xu [4 5],
Qichen Fang [1*], Yang Ye [2 3*], Weiping Jia [1*]

Supplementary Fig. S1 Total BAs in different glucometabolic status during OGTT.

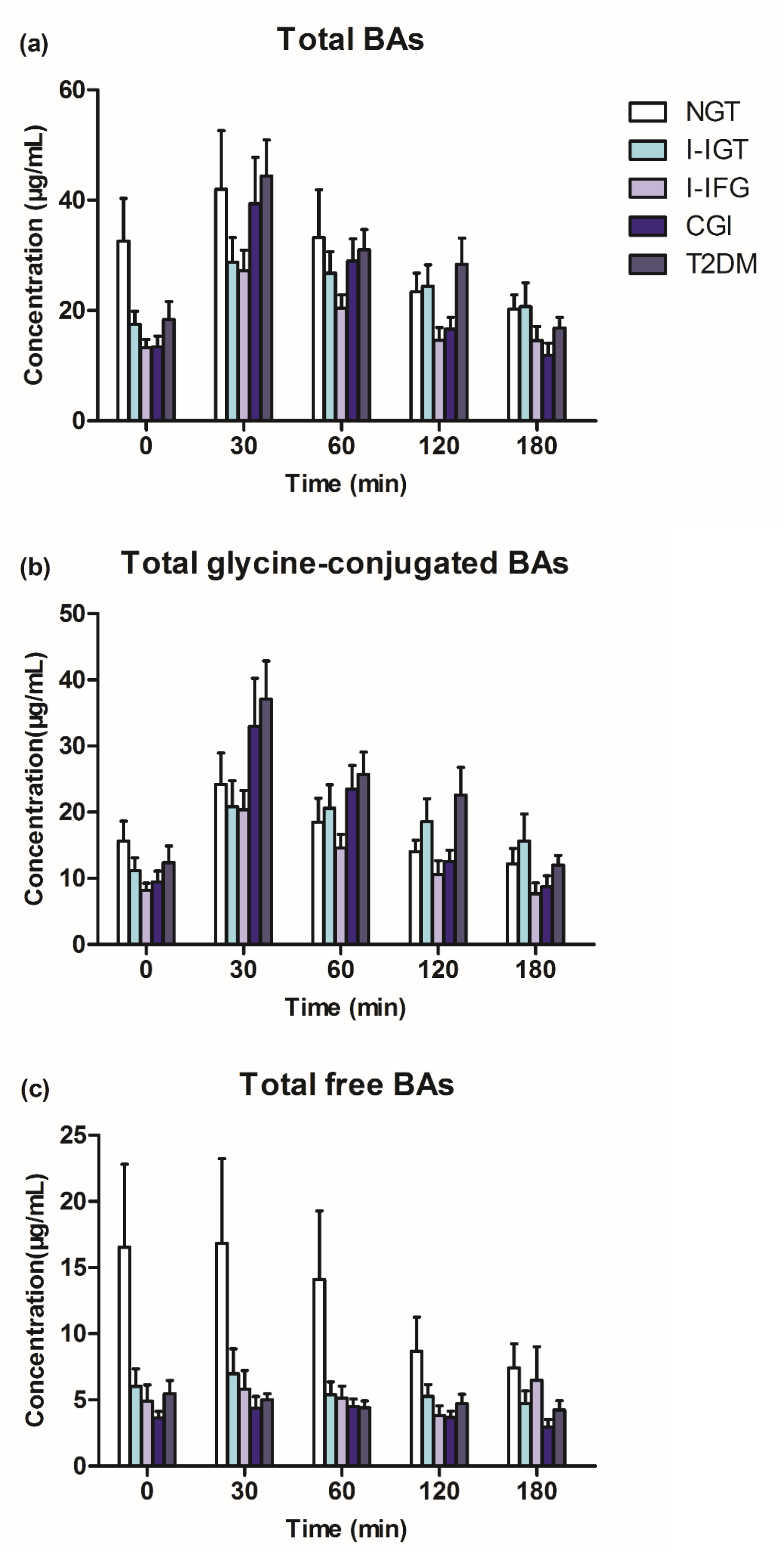

Supplementary Fig. S2 Glycine-conjugated BAs in different glucometabolic status during OGTT.

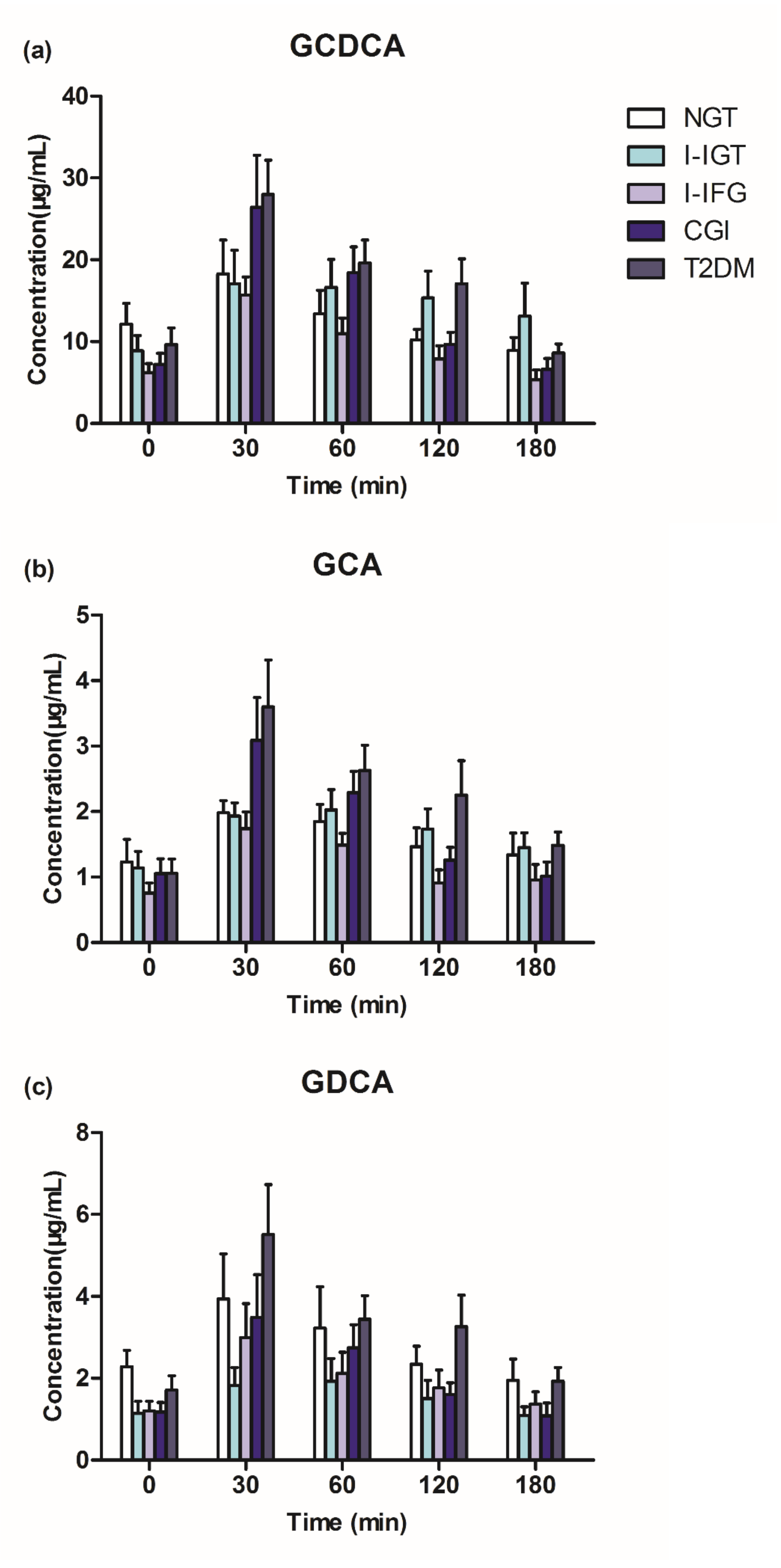

Supplementary Fig. S3 Free BAs in different glucometabolic status during OGTT.

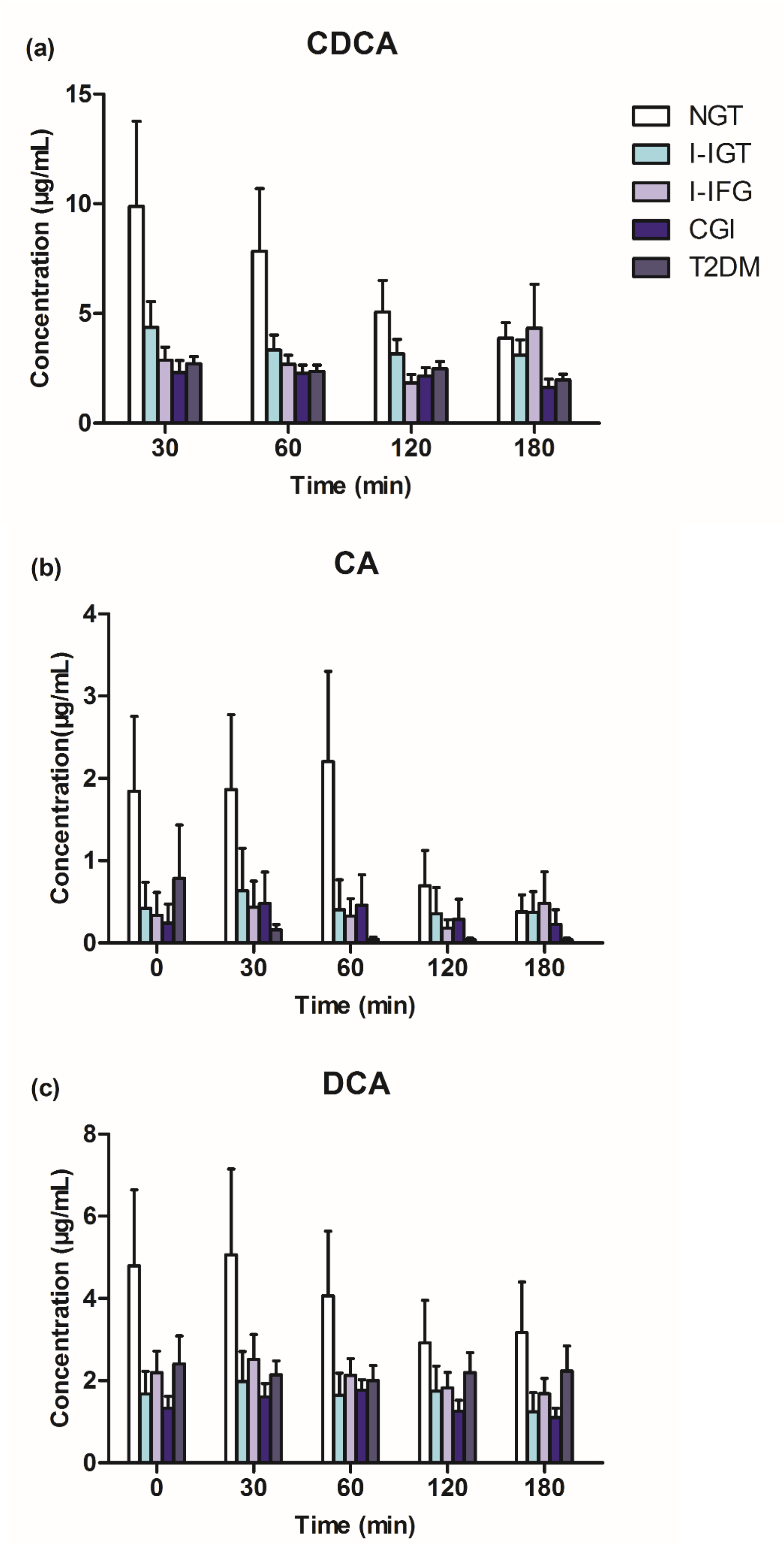

Supplementary Fig. S4 Proportions of fasting BAs in subjects with different glucose tolerance state (n=65).

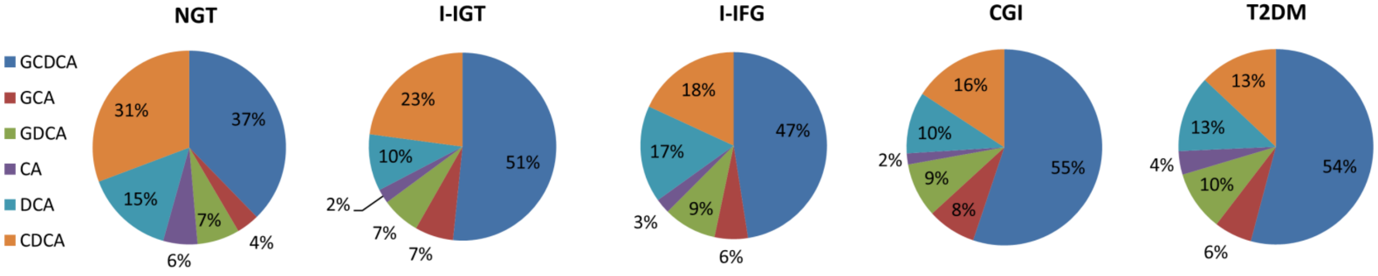

Supplementary Fig. S5 CDCA increases the effect of FGF19 in HepG2 cells. HepG2 cells were stimulated for 24h with 100 μmol/L of CDCA in the presence or absence of 10 μmol/L of FXR antagonist GS. Total RNA was prepared and FGF19 (a), PGC-1α (b) and G6Pase (c) mRNA was analyzed. Data are mean ± SEM, n=3. ****P* **< 0.05.**

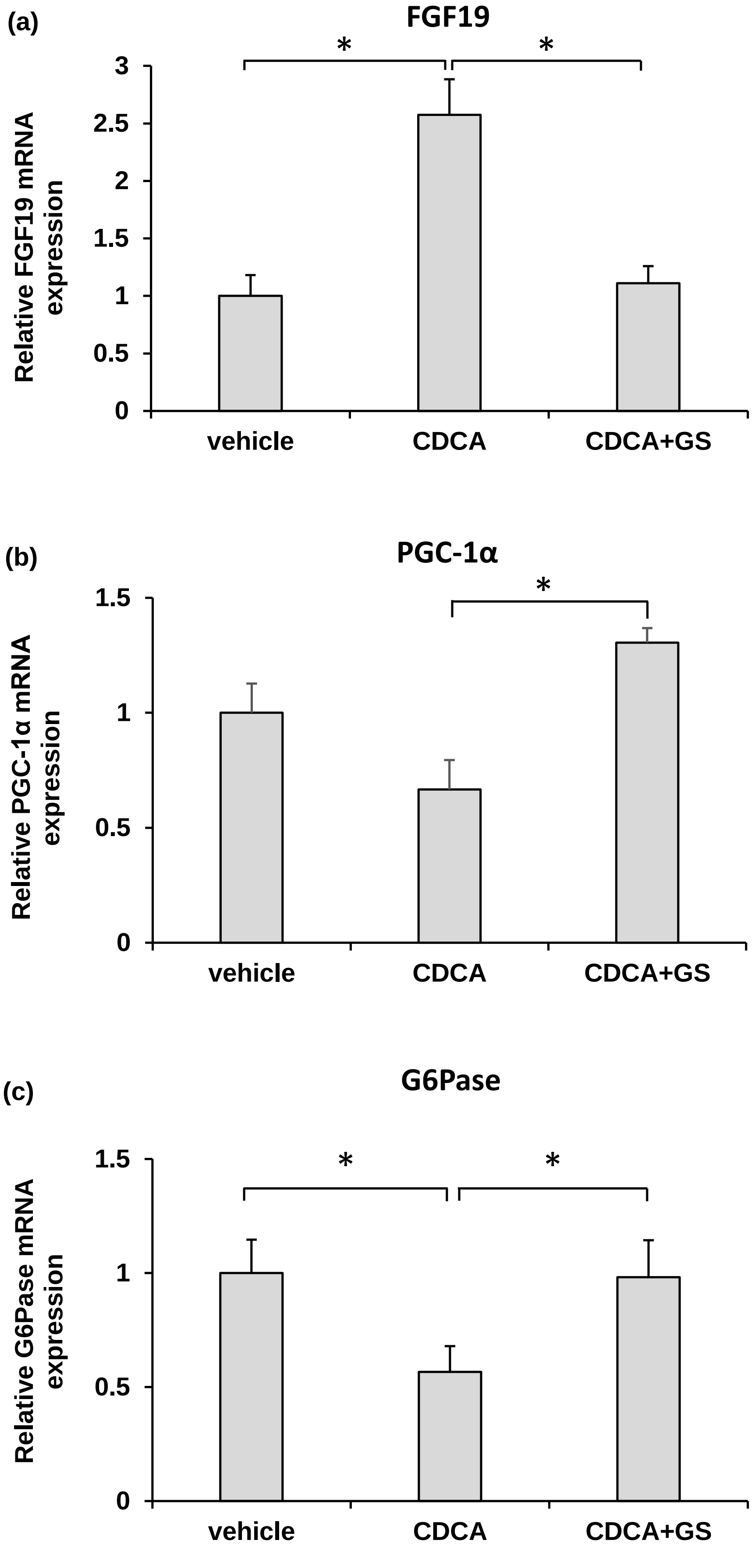

Supplement: Supplementary file 1 — supplementary figure [file 41598_2017_6252_MOESM1_ESM.pdf]
